# Multi-terrane structure controls the contrasting lithospheric evolution beneath the western and central–eastern Tibetan plateau

**DOI:** 10.1038/s41467-018-06233-x

**Published:** 2018-09-17

**Authors:** Pengpeng Huangfu, Zhong-Hai Li, Taras Gerya, Weiming Fan, Kai-Jun Zhang, Huai Zhang, Yaolin Shi

**Affiliations:** 1 Key Laboratory of Computational Geodynamics, College of Earth and Planetary Sciences, University of Chinese Academy of Sciences, 100049 Beijing, China; 2 Laboratory for Marine Geology, Qingdao National Laboratory for Marine Science and Technology, 266237 Qingdao, China; 3 Department of Earth Sciences, Institute of Geophysics, ETH-Zurich, 8092 Zurich, Switzerland; 4 CAS Center for Excellence in Tibetan Plateau Earth Sciences, 100101 Beijing, China; 5 Key Laboratory of Continental Collision and Plateau Uplift, Institute of Tibetan Plateau Research, Chinese Academy of Sciences, 100101 Beijing, China

## Abstract

The Tibetan plateau is manifested by contrasting along-strike lithospheric structures, but its formation mechanism and the relationship with the heterogeneous multi-terrane configuration is a challenging problem. Here we conduct systematic numerical modeling to explore the roles of width, density, and rheological properties of the multiple terranes in the lithospheric evolution of the Tibetan plateau, which reveals two distinct collision modes. In Mode-I, the lithospheric mantles of both the strong and weak terranes in the Tibetan plate are completely detached, followed by the underthrusting of Indian lithosphere beneath the whole plateau. Alternatively, Mode-II is characterized by full detachment of the weak terranes, but (partial) residue of the strong terranes during collision. These two contrasting modes, broadly consistent with the lithospheric structures of western and central–eastern Tibetan plateau, respectively, are strongly dependent on the along-strike variation of the width of the strong Lhasa–Qiangtang terranes.

## Abstract (web summary)

The Tibetan plateau is manifested by contrasting along-strike lithospheric structures, but the correlation with multi-terrane configuration remains challenging. Here, the authors show the crucial roles of the original geometric shape of accreted terranes in regulating the lithospheric evolution of Tibetan plateau.

## Introduction

**Fig. 1:**
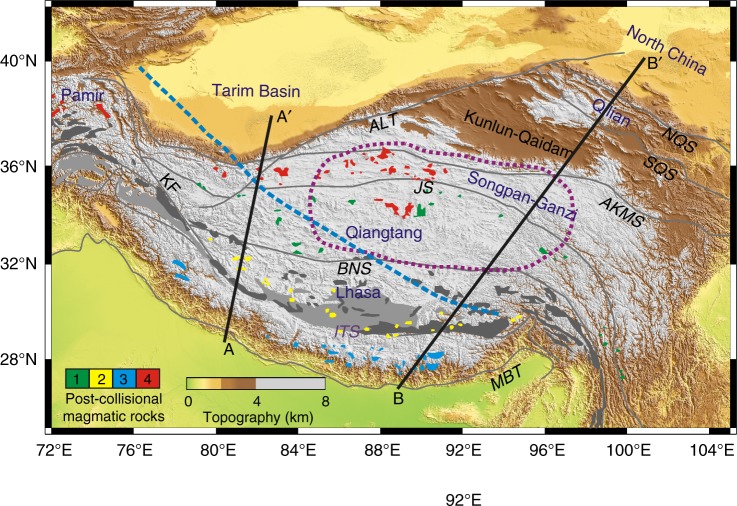
Tectonic framework of the Tibetan plateau. Four main suites of post-collisional magmatic rocks are shown with different color: 1 (green) = Eocene to Oligocene potassic rocks; 2 (yellow) = Late Oligocene to Mid-Miocene rocks; 3 (blue) = Himalayan leucogranites; 4 (red) = Mid-Miocene to Quaternary potassic rocks^21,49,65^ The dark and light gray areas in southern Tibet denote Gangdese granitoids and Paleogene Linzizong volcanic rocks, respectively^49^. The thick purple dashed line delineates the zone of poor Sn propagation^5,12–14^. The thick blue dashed line represents the northern boundary of the Indian lithosphere at 200 km depth^5^. Profiles AA′ and BB′ are locations of schematic cross-sections illustrated in Fig. 6, representing the lithospheric structure of the western and central–eastern Himalayan–Tibetan orogen. Gray lines mark the major fault traces and suture zones. MBT Main Boundary Thrust, ITS Indus–Tsangpo suture, BNS Bangong–Nujiang suture, JS Jinsha suture, AKMS Anyimaqen–Kunlun–Muztagh suture, SQS South Qilian suture, NQS North Qilian suture, KF Karakoram fault, ALT Altyn Tagh fault. The Generic Mapping Tools^66^ was used to create the topographic map, with the topography data from ETOPO1 Global Relief Model (https://www.ngdc.noaa.gov/mgg/global/global.html). In addition, the software CorelDRAW was further used to draw all the other elements in the figure

Many orogenic systems are constructed of multiple allochthonous terranes with distinct evolution histories and geometric–thermo–rheological properties^1–4^. The original heterogeneities of the accreted terranes could significantly affect post-collisional deformation behaviors and overall crustal–lithospheric structure of the collision zone. Taking the Himalayan–Tibetan orogen as an example, the Tibetan lithosphere was composed of accreted terranes before the Cenozoic India–Asia collision^3^ (Fig. 1). The major terranes, from south to north, include Lhasa, Qiangtang, Songpan–Ganzi (SPGZ henceforth), Kunlun–Qaidam, and Qilian terranes. In addition, the Tibetan plateau is also manifested by contrasting along-strike lithospheric structures from west to east^5–8^. Specifically, Indian lithosphere is supposed to horizontally underthrust the western Tibetan plateau^5^, which is presumably accompanied by southward Asian subduction in the Pamir region^9–11^. However, in central–eastern Tibet, the Indian and North China lithospheres separately underthrust southern and northeastern Tibet, with a weak lithospheric region in between, mainly beneath the Qiangtang and SPGZ terranes. This weak lithosphere is marked by high temperature, poor Sn propagation, low Pn velocities, and strikingly large seismic anisotropy^5,12–14^ (Fig. 1). The mechanism of the contrasting lithospheric structures from western to eastern Tibet and the correlation with multi-terrane configuration remain challenging.

The along-strike (west–east) rheological heterogeneities of the Tibetan lithosphere, inherited from its tectonic history, are generally invoked to account for the heterogeneous deep structure beneath the plateau^8,15^. However, it is generally neglected for the across-strike (north–south) variations of the crustal composition and rheological properties among the multiple terranes, which are generally observed^3,16^ and show contrasting characteristics between the terranes south and north of the Jinsha suture, according to the stratigraphy^16^ and pre-Cenozoic tectonic histories^3,17,18^. In addition, the present Tibetan plateau displays an eastward-broadening shape, which might reflect the potential geometry of the Tibetan lithosphere at the onset of the India–Asia collision. Given the above-mentioned along-strike and across-strike heterogeneities, it is thus interesting to explore whether and how the intrinsically heterogeneous multi-terrane configuration of the Tibetan lithosphere contributes to the along-strike variations of the lithospheric structure beneath the plateau. The answer to this question is crucial to better understand the dynamics of the India–Asia collision, as well as the development of the Tibetan plateau.

Previous studies revealed that the properties of accreted terranes and the sutures among them can strongly affect the evolution of continental orogens, such as the first-order tectonic structure^19^, crustal/lithospheric deformation behavior^20^, and spatial–temporal patterns of magmatism^19,21,22^. Numerical models, together with geological and geophysical observations, can test the hypotheses for the tectonic evolution of the Tibetan plateau, including distributed crustal shortening and thickening^23^, lateral extrusions along major strike-slip faults^24^, Indian lithosphere underthrusting^25^, and crustal channel flow^26,27^. Most previous numerical studies regarding allochthonous terranes focused on the terrane accretion processes^28–30^, rather than the terminal continental collision and mountain building^25,31^. For the latter cases, Kelly et al.^25^ integrated one soft accreted (Lhasa) terrane in the models, focusing on the roles of the soft Lhasa terrane in tectonic evolution of southern Tibet. Li et al.^31^ conducted similar models, although the weak terrane, sandwiched between the relatively strong Indian and Asian plates, was interpreted as the whole Tibetan lithosphere, rather than only the Lhasa terrane in Kelly et al^25^. Both independent studies obtained similar conclusions, highlighting the prerequisite of a relatively dense and weak accreted terrane for further delamination/detachment during convergence, which may be followed by the sub-horizontal underthrusting of Indian lithosphere (see Supplementary Note 1 for details). In all other previous numerical studies, the Tibetan lithosphere was generally simplified to a homogeneous continental plate without considering the above-mentioned, significant multi-terrane heterogeneity, thus neglecting the reactivation of pre-existing sutures and its effects on the Indian–Asian collision.

In this study, we perform systematic numerical experiments to explore the effects of different geometric–mechanical properties of multiple terranes on the post-collisional lithospheric deformation, which provides crucial implications for the role of multi-terrane configuration in forming the contrasting along-strike lithospheric structures of the Tibetan plateau. The model results are further constrained by post-collisional magmatism and deep mantle tomography, which indicate that the width of relatively stronger Lhasa–Qiangtang terranes is a key controlling factor for the geodynamic evolution of the Tibetan plateau.

## Results

### Model setup

**Fig. 2:**
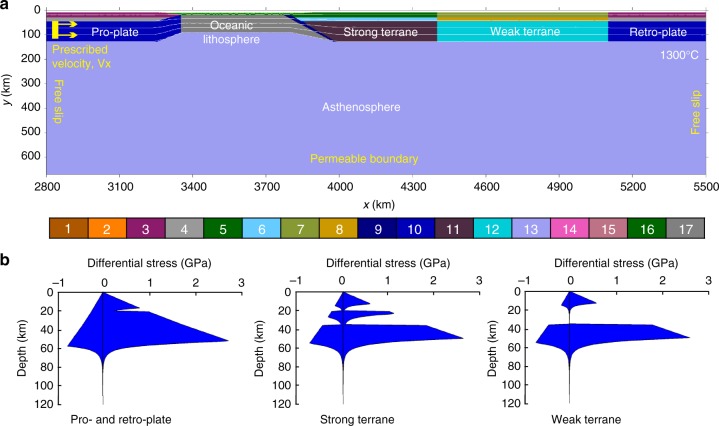
Model setup. **a** Initial model configuration. Enlargement (2700 × 670 km) of the numerical box (6400 × 670 km) is shown. White lines are isotherms with an interval of 300 °C. A constant velocity of 5 cm yr^−1^ is assigned within the pro-plate. Different colors refer to different lithologies, with the colorbar: 1/2 = sediment; 3 = pro-/retro-plate upper crust; 4 = pro-/retro-plate lower crust; 5 = strong terrane upper crust; 6 = strong terrane lower crust; 7 = weak terrane upper crust; 8 = weak terrane lower crust; 9 = initial weak zone; 10 = pro-lithospheric and retro-lithospheric mantle; 11 = strong terrane lithospheric mantle; 12 = weak terrane lithospheric mantle; 13 = asthenosphere; 14 = partially molten upper crust of the pro-plate and retro-plate; 15 = partially molten upper crust of the strong and weak terranes; 16 = oceanic crust; 17 = oceanic lithospheric mantle. **b** Three rheological structures of the continental lithosphere. Rheological strength profiles are obtained for the 120-km-thick lithosphere with an initial Moho temperature of 400 °C for pro-plate and retro-plate and of 450 °C for the strong and weak terranes. The prescribed constant strain rate is 10^−16^ s^−1^

We performed two-dimensional (2D) thermomechanical numerical experiments (see Methods) to investigate the correlation of post-collisional crustal–lithospheric structure with the across-strike multi-terrane configuration in collision zones. With respect to the Tibetan plateau, we divided all the terranes constituting the plateau into two composite terranes bordered by the Jinsha suture, according to the stratigraphy^16^ and pre-Cenozoic tectonic histories^3,17,18^. Specifically, the Lhasa and Qiangtang terranes can be regarded as an integrated strong terrane, since both terranes are underlain by the Precambrian crystalline basement that was overlapped by Pan-African metamorphism and magmatism and then covered by similar Paleozoic marine strata^16,32^. It is generally believed that these two terranes were part of the northern Gondwana margin during most of the Paleozoic^17^, and separated in the Permian^3,33^ or the early Mosozoic^17,34^. However, the terranes north of the Jinsha suture, including the SPGZ, Kunlun–Qaidam, and Qilian terranes, are distinct from the Lhasa and Qiangtang terranes during Paleozoic–Mesozoic times in terms of faunal affinities^33^, detrital zircon ages^17^, and tectonic evolution^18^. Multiple sutures exist in this region^18,35–37^, which indicates that the northern Tibet was a locus of long-lived oceanic subduction and repeated micro-continent/arc–micro-continent collision, followed by orogen collapse-induced rifting or even oceanic spreading since the Neoproterozoic^18^. In combination with the widespread, thick flysch sediments deposited in the SPGZ terrane^3^, intense Cenozoic deformation^38^, and the widely distributed nature of active thrusts in northeastern Tibet^20^, these terranes north of the Jinsha suture are generally considered to be weaker than the southern terranes. Therefore, as a first-order assumption, we integrate the Lhasa and Qiangtang terranes as a composite strong terrane, and all the terranes north of the Jinsha suture as a composite weak terrane in the numerical models (Fig. 2a), in order to reveal the effects of the multi-terrane heterogeneity. Although the two-integrated-terrane configuration is a simplified tectonic structure, it reflects the most fundamental rheological characteristics of the accreted terranes in the Tibetan lithosphere.

A 500-km Neo-Tethyan oceanic lithosphere is also included in the initial model (Fig. 2a), which, together with the two integrated continental terranes, is located between the pro-plate (i.e., Indian plate) and retro-plate (i.e., Tarim–North China plate) (Fig. 2a). Three types of lithospheric rheological profiles are designed for the pro-/retro-plate, the strong terrane, and the weak terrane (Fig. 2b). Detailed properties of different rock types are described in the Supplementary Tables 1, 2.

In this study, a series of numerical models are constructed with variable width of the strong and weak terranes, as well as variable reference density of their lithospheric mantles. The width of the weak terrane ranges from 400 to 700 km, whereas the strong terrane from 600 to 1200 km. According to the secular evolution from depleted Mg-rich low-density Archean mantle to more fertile, denser Phanerozoic mantle^39^, the reference density (i.e., at the same pressure and temperature) of the depleted pro-plate and retro-plate lithospheric mantle is assigned to be −30 kg m^−3^ lower than that of the asthenosphere. However, the reference density of the accreted terranes’ lithospheric mantle is assumed to be equal to the asthenosphere in the reference models. We also built two other sets of models with slightly higher (+10 kg m^−3^) or lower (−10 kg m^−3^) reference density of the accreted terranes, in order to study the effects of lithospheric density on the deformation behaviors of the terranes during collision.

### Two end-member modes controlled by the strong terrane width

**Fig. 3:**
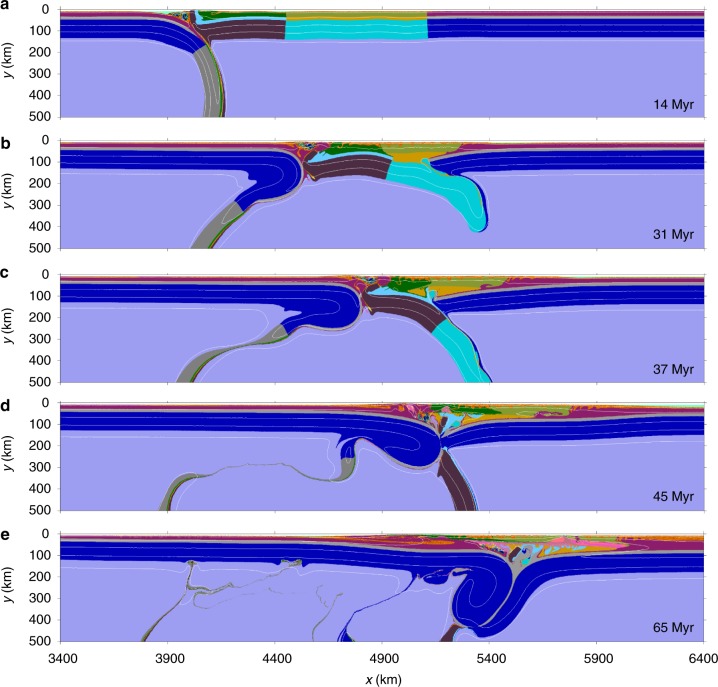
Evolution of complete terrane detachment mode (Mode-I). The widths of the strong and weak terranes are 600 and 700 km, respectively. The reference density of the lithospheric mantle of both the strong and weak terranes is the same as the asthenosphere. Time of convergence is shown in each panel. Colors of rock types are as in Fig. 2a. White numbered lines are isotherms plotted every 300 °C starting from 100 °C

**Fig. 4:**
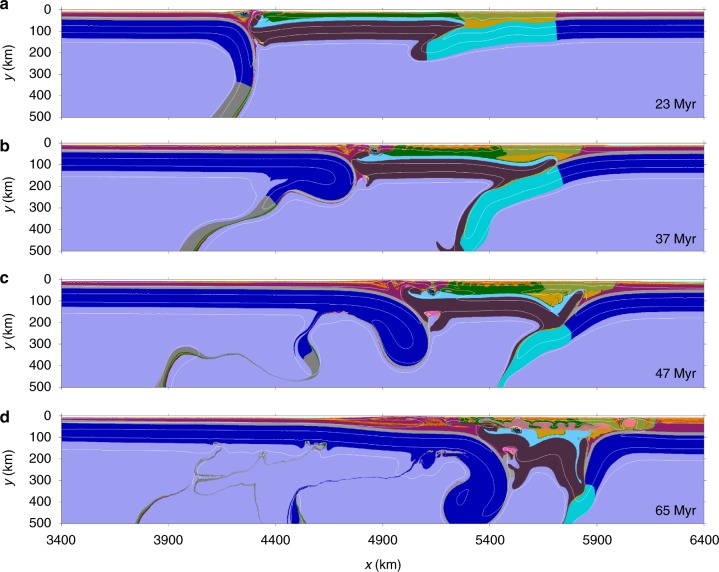
Evolution of partial terrane detachment mode (Mode-II). The widths of the strong and weak terranes are 1200 and 700 km, respectively. The reference density of the lithospheric mantle of both the strong and weak terranes is the same as the asthenosphere. Time of convergence is shown in each panel. Colors of rock types are as in Fig. 2a. White numbered lines are isotherms plotted every 300 °C starting from 100 °C

According to the deformation behaviors of the accreted terranes, the model results with neutral reference density between the terrane lithospheric mantle and the asthenosphere can be summarized into two contrasting modes as shown in Figs. 3, 4. Mode-I is characterized by complete detachment of the lithospheric mantle of both the strong and weak terranes, thus termed as “complete terrane detachment” mode. In Mode-II, the lithospheric mantle of the weak terrane is fully detached; however, that of the strong terrane is (partially) preserved in the collision zone, which is thus defined as “partial terrane detachment” mode.

Mode-I is marked by “complete terrane detachment” (Fig. 3). In the stage of oceanic subduction, only the leading end of the upper plate close to the suture is slightly deformed in the compressional regime, and the deformation of the whole overriding terranes is negligible (Fig. 3a). When continental collision begins, continuous convergence between the pro-plate and retro-plate is firstly accommodated by the localized deformation in the overriding weak terrane. Consequently, the crust of the weak terrane is scraped off and accreted onto the neighboring strong terrane and retro-plate, whereas the lithospheric mantle is detached and sinks into the asthenosphere (Fig. 3b). Afterwards, the shortening of the strong terrane takes place, with its lithospheric mantle detached following that of the weak terrane, resulting in complete lithospheric detachment of both terranes (Fig. 3c, d). Finally, the advancing pro-plate sub-horizontally underthrusts and meets the retro-plate beneath the thickened crust of the detached terranes (Fig. 3e).

Mode-II is characterized by “partial terrane detachment” (Fig. 4). After oceanic subduction (Fig. 4a), the weak terrane begins to subduct beneath the strong terrane in response to the far-field effect of continental collision, which further leads to the detachment of its lithospheric mantle (Fig. 4b, c). Subsequently, the pro-plate and retro-plate subduct beneath the residual strong terrane with opposite directions, forming a double-sided continental subduction scenario (Fig. 4c, d). It is worth noting that the retro-plate subduction is limited, comparing to that of the pro-plate, which is consistent with the geophysical observations^40^. During these processes, the intense upwelling occurs in the confined region beneath the residue strong terrane, with bottom erosion and rheological weakening of the overriding lithosphere. Continued convergence causes further subduction of both the pro-plate and retro-plate, shortening deformation of the residue terrane, and significant crustal thickening with intense partial melting in the middle crust (Fig. 4d).

**Fig. 5:**
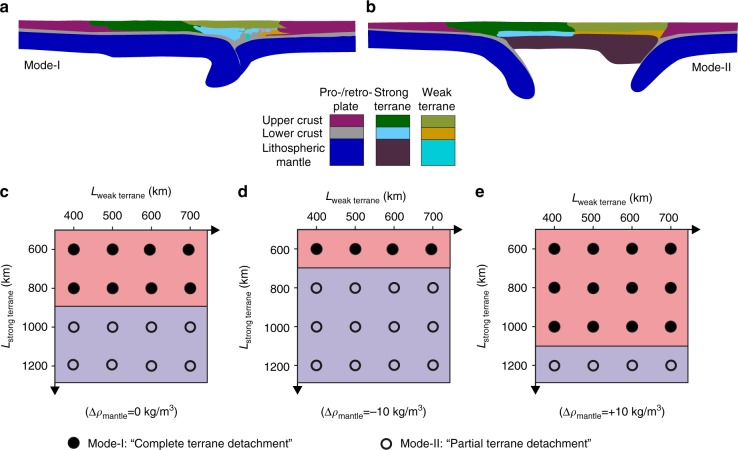
Phase diagram of the contrasting detachment modes. **a** Mode-I of complete terrane detachment, in which the lithospheric mantles of both the strong and weak terranes are detached during collision. **b** Mode-II of partial terrane detachment, in which only the weak terrane is detached, whereas the strong terrane is strongly deformed but not detached. **c** Regime diagram with the same reference density (i.e., at the same pressure and temperature) of the terrane lithospheric mantle and the asthenosphere. **d** Regime diagram with the reference density of lithospheric mantle of both terranes decreased to be −10 kg m^−3^ lower than the asthenosphere. **e** Regime diagram with the reference density of lithospheric mantle of both terranes increased to be +10 kg m^−3^ higher than the asthenosphere

The model results reveal that the contrasting modes are mainly dependent on the width of the strong terrane, whereas that of the weak terrane does not change the general model predictions (Fig. 5c). Specifically, if the strong terrane is narrow (≤800 km), complete detachment of both the weak and strong terranes is predicted. However, the strong terrane cannot be fully detached, when it becomes wider (≥1000 km). From the mechanical perspective, the contrasting modes are mainly attributed to the degree of far-field collision effect on the weak terrane. When the strong terrane is narrow, the compressional stress due to the collision can be readily transferred to retro-side (right side) of the weak terrane, which thus enables the subduction of the weak terrane beneath the retro-plate. This leads to the subsequent subduction of the strong terrane, eventually resulting in the complete detachment of both terranes. However, a relatively wider strong terrane considerably decreases the far-field effect of the collision on the weak terrane. In this case, the deformation tends to localize in the pro-side (left side) of the weak terrane, rather than the retro-side. Thereby, it appears easier for the weak terrane to subduct leftward beneath the strong terrane rather than rightward beneath the retro-plate. In this case, the subduction and detachment of strong terrane is prohibited.

### Dependence on the density of terrane lithospheric mantle

The lithospheric density is another critical factor that may control the patterns of lithosphere detachment in collisional orogens, which is further tested with both lower and higher densities. If the reference density of lithospheric mantle of both the strong and weak terranes is decreased to be −10 kg m^−^^3^ lower than the asthenosphere, Mode-I with complete detachment of both terranes is only predicted in the models with a narrower strong terrane of 600 km (Fig. 5d). Alternatively, if the reference density of lithospheric mantle of both the strong and weak terranes is increased to be +10 kg m^−3^ higher than the asthenosphere, Mode-I with complete terrane detachment develops more extensively in the models with a larger range of strong terrane width (≤1000 km) (Fig. 5e). It indicates that the lithospheric density also strongly affects the detachment modes of the accreted terranes. A larger positive density contrast between the terrane and asthenosphere promotes the detachment during collision, and vice versa.

## Discussion

**Fig. 6:**
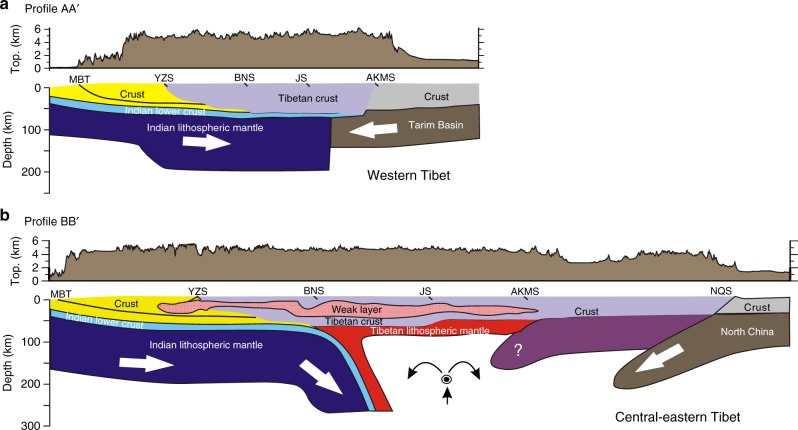
Cross-section showing lithospheric structures of the Tibetan plateau. **a** The integrated schematic cross-section in western Tibet. The lithospheric structure is derived from the seismic data^5,6^; the crustal tectonic structure is from Searle et al.^67^ and Yin and Harrison^3^. **b** The integrated schematic cross-section in central–eastern Tibet, which is modified after Owens and Zandt^13^ and Ye et al.^40^. The weak layer in the middle crust is after Wang et al.^68^. The mantle flow underneath central and northern Tibet is supposed to be eastward^69^. The locations of both profiles are marked in Fig. 1

Various geophysical observations suggest contrasting lithospheric structures beneath the Tibetan plateau^5–7,41^. On the basis of these previous studies, we assemble two distinct schematic interpretations of the present-day lithospheric structural configuration, representing the western and central–eastern Tibetan plateau (Fig. 6). Previous geophysical studies of the entire Himalayan–Tibetan collision zone show systematic along-strike variations of the architecture of the Indian underthrusting^5,6,8,41^. Firstly, the horizontal distance over which the Indian lithosphere underthrusts the plateau decreases from west to east. The Indian lithosphere underlies the entire plateau in western Tibet, whereas it roughly reaches the Bangong–Nujiang suture in central Tibet, or subducts steeply around the Indus–Tsangpo suture in eastern Tibet^6,42^. Secondly, the dip angle of the leading end of the subducting Indian slab decreases from east to west^8^. In eastern–central Tibet, the leading edge of the Indian lithosphere steeply plunges into the mantle beneath the plateau, but in western Tibet, the Indian lithosphere is suggested to horizontally underthrust the plateau without significant sinking. Similar features are also captured in the two contrasting modes as shown in Fig. 5a, b, which reveal their strong dependence on the lithospheric deformation/detachment of the overriding Lhasa–Qiangtang terranes.

In order to obtain the lithospheric structure of western Tibet, the lithospheric mantles of the accreted terranes are required to be gradually detached during the India–Asia collision and further replaced by the cold Indian lithosphere, with a process similar to that shown in Fig. 3. These geodynamic processes not only constrain the fate of the terranes’ lithospheric mantle in western Tibet, but also account for the development of the plateau there. It is worth noting that the evolution of long-distance continental underthrusting in the numerical models (Fig. 3) indicates a similar dynamic process of Indian underthrusting beneath the Pamir–West Tibet as demonstrated in previous studies^5,10,11^. The Moho discontinuity lies at depths of about 60–70 km in this area^5^, which is also consistent with the “complete terrane detachment” mode (Fig. 3e). In addition, the numerical models suggest that the crustal thickening is driven by the combined effects of previous crustal shortening of the accreted terranes and subsequent underthrusting and duplexing of the Indian lower crust (Fig. 3).

In the model with complete terrane detachment (Fig. 3), the underthrusted cold Indian lithosphere beneath the collision zone may lead to relatively low thermal condition of the orogen, thus inhibiting large-scale magmatism. This agrees with magmatism in the western Tibetan plateau where the distribution of post-collisional volcanic rocks is limited. The main magmatic episode there is in the early Miocene^43^, which is supposed to be induced by the detachment of accreted terranes prior to the underthrusting of Indian lithosphere beneath the collision zone^10^, thus consistent with our model predictions (Fig. 3c, d).

In central–eastern Tibet (roughly east of 82°E), the Indian lithosphere underthrusts the plateau probably to the Bangong–Nujiang suture or more southern^5,6^, where the Indian plate steeply subducts to the asthenosphere (Fig. 6b). In addition, the underthrusting of the North China plate beneath northeastern Tibet^40^, together with the northward subduction of the Indian plate, jointly forms a complex lithospheric structure beneath the broad plateau similar to the “partial terrane detachment” mode (cf. Figs. 4d, 6b). The proceeding lithospheric thinning in northern Tibet, that is, the zone of poor Sn propagation^5,12–14^ denoted by the purple dashed line in Fig. 1, is presumably due to the upwelling of the partially molten materials from the subducting Indian slab (Fig. 4). On the other hand, the southward subduction of the Qaidam basin is still a widely debated issue (Fig. 6b). Previous receiver function images revealed the subduction of the Qaidam basin beneath northern Tibet from 100 km depth at the Kunlun fault to about 200 km depth in the Qiangtang terrane^5,44,45^, which appears to be supported by crustal shortening and northeastern propagation of thrust fault systems in northeastern Tibet^19,46,47^. However, this assumed subduction was not observed in a latest tomographic model based on a 3D adjoint tomography method^48^. Lithospheric deformation of the central–eastern Tibetan plateau is nevertheless primarily attributed to the subduction of Indian and Asian plates. The “partial terrane detachment” mode is thus well constrained by this first-order lithospheric structure, while the absence of Qaidam basin in this study is due to the model simplifications.

The orogenic process illustrated in our models (Fig. 4) can be further supported by the spatial–temporal distribution of post-collisional magmatism in the broad central–eastern Tibet (Fig. 1), where four main magmatic episodes^49^ can be recognized from the early Eocene to the Quaternary. Firstly, widespread potassium-rich lavas erupted in the Qiangtang terrane from the Eocene to Oligocene^50^. Then the magmatism migrated southwards, with adakitic and ultrapotassic lavas from late Oligocene to mid-Miocene in the Lhasa terrane^51^, accompanying the Miocene Himalayan leucogranites derived from the melting of Indian crust^52^. In the final episode, extensive potassic volcanism resumed since the mid-Miocene in the northern plateau^53^. Based on the numerical models (Fig. 4), the first magmatic episode was related to the subduction and further detachment of the weak terrane beneath the Qiangtang terrane as a consequence of the far-field India–Asia convergence. The second episode with adakitic and ultrapotassic characteristics may be attributed to the Indian subduction beneath the Lhasa terrane. Eventually, asthenospheric upwelling beneath the residue terranes not only caused obvious lithospheric thinning in the central Tibet, but also triggered extensive magmatism there since the Mid-Miocene (Fig. 4d).

**Fig. 7:**
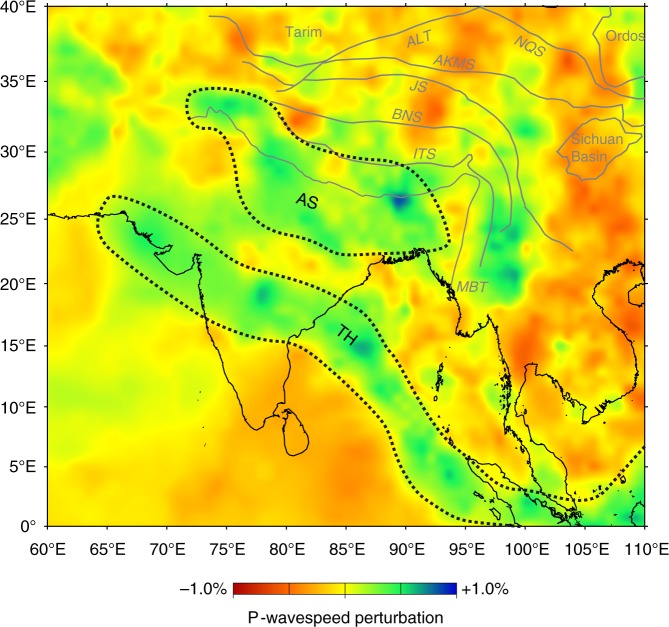
Tomographic section at 1016 km depth suggesting two previous detachment events. Two high-speed anomalies, denoted as TH and AS, are observed. On the basis of their geometry and position, the TH anomaly is interpreted as the detached Neo-Tethyan oceanic slab^54,55^, whereas the AS anomaly might be the signature of the detached Tibetan lithospheric mantle during the India–Asia collision^55^. The Generic Mapping Tools^66^ was used to create the map, with the open source dataset of P-wave speed variations from Li et al.^70^.

Notably, there are two positive anomalies beneath the Indian–Tibetan plateau area in the tomographic images at 1016 km depth, which are denoted as TH and AS in Fig. 7. According to our numerical models, the elongated “TH” anomaly can be regarded as the remnant Neo-Tethyan oceanic slab^54,55^, which was detached from the Indian plate shortly after the collision and is now locating dramatically south of the Indus–Tsangpo suture. In contrast, the AS anomaly can be interpreted as the signature of the detached lithospheric mantle of the accreted terranes^55^. These interpretations are consistent with previous geophysical observations^55^ and post-collisional volcanism^49^.

To conclude, the geodynamic evolution of the Himalayan–Tibetan orogen could be the combined processes of complete terrane detachment mode in western Tibet and partial terrane detachment mode in central–eastern Tibet, which are strongly controlled by the width and properties of the relatively strong Lhasa–Qiangtang terranes. The comparisons between the model results and geological/geophysical observations further suggest that the strong terranes might be originally narrower in western Tibet than in central–eastern Tibet at the onset of the India–Asia collision. It is reasonable, because (1) the present-day eastward-broadening shape of the Tibetan plateau probably inherited a similar geometric shape of the Lhasa–Qiangtang terranes in the incipient collision, and (2) the analyses of fold-and-thrust belts indicate that the Cenozoic shortening of central–eastern Lhasa–Qiangtang terranes is generally larger than that of the western terranes^3,20^.

## Methods

### Numerical code and governing equations

The numerical experiments were performed using a finite-difference numerical code (I2VIS) with marker-in-cell technique^56^. The code solves the momentum, continuity, and heat conservation equations for a 2D creeping flow including thermal and chemical buoyant forces, that is,

2D stokes equations:

1 $$\begin{array}{*{20}{c}} {\frac{{\partial {\mathbf{\sigma }}_{xx}^\prime }}{{\partial x}} + \frac{{\partial {\mathbf{\sigma }}_{xz}^\prime }}{{\partial z}} = \frac{{\partial P}}{{\partial x}}} \\ {\frac{{\partial {\mathbf{\sigma }}_{zx}^\prime }}{{\partial x}} + \frac{{\partial {\mathbf{\sigma }}_{zz}^\prime }}{{\partial z}} = \frac{{\partial P}}{{\partial z}} - g\rho \left( {C,M,P,T} \right)}, \end{array}$$

where *x* and *z* are, respectively, horizontal and vertical coordinates, *g* is gravitational acceleration, ${\mathbf{\sigma }}_{ij}^\prime$ are components of deviatoric stress tensor, and the density *ρ* depends on composition (*C*), melt fraction (*M*), temperature (*T*), and pressure (*P*).

Incompressible continuity equation:

2 $$\frac{{\partial {\mathbf{v}}_x}}{{\partial x}} + \frac{{\partial {\mathbf{v}}_z}}{{\partial z}} = 0,$$

where **v**_*x*_ and **v**_*z*_ are velocity components.

Heat conservation equations:

3 $$\begin{array}{*{20}{c}} {\rho C_{\mathrm{p}}\left( {\frac{{\mathrm{D}T}}{{\mathrm{D}t}}} \right) = - \frac{{\partial {\mathbf{q}}_x}}{{\partial x}} - \frac{{\partial {\mathbf{q}}_z}}{{\partial z}} + H_{\mathrm{r}} + H_{\mathrm{a}} + H_{\mathrm{s}}}, \\ {{\mathbf{q}}_x = - k\left( {T,P,C} \right)\frac{{\partial T}}{{\partial x}}\quad {\mathbf{q}}_z = - k\left( {T{\mathrm{,}}P,C} \right)\frac{{\partial T}}{{\partial z}}}, \\ {H_{\mathrm{a}} = T\alpha \frac{{{\mathrm{d}}P}}{{{\mathrm{d}}t}}\quad H_{\mathrm{s}} = {\mathbf{\sigma }}_{xx}^\prime {\dot{\mathbf \varepsilon }}_{xx} + {\mathbf{\sigma }}_{zz}^\prime {\dot{\mathbf \varepsilon }}_{zz} + 2{\mathbf{\sigma }}_{xz}^\prime {\dot{\mathbf \varepsilon }}_{xz}}, \end{array}$$

where *C*_p_ is the effective isobaric heat capacity, D*T*/D*t* is the substantive time derivative of temperature, **q**_*x*_ and **q**_*z*_ are heat flux components, *H*_r_, *H*_a_, and *H*_s_ denote radioactive heat production, the energetic effect of isothermal (de)compression (i.e., adiabatic heating/cooling), and shear heating, respectively, *k*(*T*,*P*,*C*) is the thermal conductivity as a function of temperature, pressure, and composition^57^, *α* is thermal expansion coefficient, and ${\dot{\mathbf \varepsilon }}_{ij}$ is the strain rate tensor.

### Rheological model

The relationship between the deviatoric stress (${\mathbf{\sigma }}_{ij}^\prime$) and the strain rate (${\dot{\mathbf \varepsilon }}_{ij}$) tensors is described by realistic visco-plastic constitutive laws. In case of incompressible viscous deformation, the viscous law of friction is:

4 $$\begin{array}{*{20}{c}} {{\mathbf{\sigma }}_{xx}^\prime = 2\eta _{{\mathrm{eff}}}{\dot{\mathbf \varepsilon }}_{xx}}, & {{\dot{\mathbf \varepsilon }}_{xx} = \frac{{\partial {\mathbf{v}}_x}}{{\partial x}}}, \\ {\sigma _{xz}^\prime = 2\eta _{{\mathrm{eff}}}{\dot{\mathbf \varepsilon }}_{xz}}, & {{\dot{\mathbf \varepsilon }}_{xz} = \frac{1}{2}\left( {\frac{{\partial {\mathbf{v}}_x}}{{\partial z}} + \frac{{\partial {\mathbf{\nu }}_z}}{{\partial x}}} \right)}, \\ {{\mathbf{\sigma }}_{zz}^\prime = 2\eta _{{\mathrm{eff}}}{\dot{\mathbf \varepsilon }}_{zz}}, & {{\dot{\mathbf \varepsilon }}_{zz} = \frac{{\partial {\mathbf{v}}_z}}{{\partial z}}}, \end{array}$$

where *η*_eff_ is the effective viscosity which depends on pressure, temperature, composition, strain rate, and degree of melting.

For rocks containing small melt fractions (*M* *<* 0.1, *M* is the volumetric melt fraction), the effective viscosity for ductile creep (*η*_ductile_) as a function of pressure, temperature, composition, and strain rate invariant is defined by:

5 $$\eta _{{\mathrm{ductile}}} = \left( {{\dot{\mathbf \varepsilon }}_{{\mathrm{II}}}} \right)^{\frac{{1 - n}}{n}}\left( {A_{\mathrm{D}}} \right)^{ - \frac{1}{n}}\exp \left( {\frac{{E + PV}}{{nRT}}} \right),$$

where ${\dot{\mathbf \varepsilon }}_{{\mathrm{II}}} = \left( {0.5{\dot{\mathbf \varepsilon }}_{ij}{\dot{\mathbf \varepsilon }}_{ij}} \right)^{1/2}$ is the second invariant of the strain rate tensor, and *n*, *A*_D_, *E*, and *V* are experimentally determined flow law parameters (Supplementary Table 1), which represent stress exponent, material constant, activation energy, and activation volume, respectively. For rocks containing large melt fractions (*M* ≥ 0.1, *M* is the volumetric melt fraction), the effective viscosity of partial molten rocks is assigned to a low value of 10^18^ Pa s, that is, the minimum cut-off value of the effective viscosity.

The ductile rheology is combined with a brittle/plastic rheology to yield an effective visco-plastic rheology. For this purpose, the extended Drucker–Prager yield criterion^58^ is implemented as follows:

6 $$\begin{array}{*{20}{c}} {\sigma _{{\mathrm{yield}}} = C_0 + P{\mathrm{sin}}\left( {\varphi _{{\mathrm{eff}}}} \right)}, \\ {\sin \left( {\varphi _{{\mathrm{eff}}}} \right) = \sin \left( \varphi \right)\left( {1 - \lambda } \right)}, \\ {\eta _{{\mathrm{plastic}}} = \frac{{\sigma _{{\mathrm{yield}}}}}{{2{\dot{\mathbf \varepsilon }}_{{\mathrm{II}}}}}}, \end{array}$$

where *σ*_yield_ is the yield stress, *C*_0_ is the cohesion, *φ* is the internal frictional angle, *P* is the dynamic pressure, *φ*_eff_ can be illustrated as the effective internal frictional angle that integrates the effects of the initial frictional angle (*φ*) and pore fluid coefficient (*λ*)^59^, and ${\dot{\mathbf \varepsilon }}_{{\mathrm{II}}}$ is the second invariant of the strain rate tensor. In this paper, the plastic rheology is implemented by variable values of sin(*φ*_eff_) for different rock types (Supplementary Table 2).

With the *η*_ductile_ and *η*_plastic_, visco-plastic rheology is assigned to the model by means of a Christmas tree-like criterion, where the rheological behavior depends on the minimum viscosity attained between the ductile and brittle/plastic fields^58^, which is further controlled by the cut-off values of (10^18^–10^25^ Pa s):

7 $$\eta _{{\mathrm{creep}}} = {\mathrm{min}}\left( {\eta _{{\mathrm{ductile}}},\eta _{{\mathrm{plastic}}}} \right).$$

### Partial melting

The numerical code accounts for partial melting of the various lithologies using experimentally obtained *P*–*T* dependent wet solidus and dry liquidus curves. Volumetric melt fraction *M* is assumed to increase linearly with temperature according to the following relations^60^:

8 $$\begin{array}{*{20}{c}} {M = 0,\quad{\mathrm{when}}\,T \le T_{{\mathrm{solidus}}}}, \\ {M = \frac{{\left( {T - T_{{\mathrm{solidus}}}} \right)}}{{\left( {T_{{\mathrm{liquidus}}} - T_{{\mathrm{solidus}}}} \right)}},\quad{\mathrm{when}}\,T_{{\mathrm{solidus}}} < T < T_{{\mathrm{liquidus}}}}, \\ {M = 1,\quad{\mathrm{when}}\,T \ge T_{{\mathrm{liquidus}}}}, \end{array}$$

where *T*_solidus_ and *T*_liquidus_ are the wet solidus and dry liquidus temperature of the given lithology (Supplementary Table 2), respectively.

Consequently, the effective density (*ρ*_eff_) of partially molten rocks varies with the amount of melt fraction and *P–T* conditions according to the relation:

9 $$\rho _{{\mathrm{eff}}} = \rho _{{\mathrm{solid}}} - M\left( {\rho _{{\mathrm{solid}}} - \rho _{{\mathrm{molten}}}} \right),$$

where *ρ*_solid_ and *ρ*_molten_ are the densities of the solid and molten rock, respectively, which change with pressure and temperature according to the relation:

10 $$\rho _{P,T} = \rho _0\left[ {1 - \alpha \left( {T - T_0} \right)} \right]\left[ {1 + \beta \left( {P - P_0} \right)} \right],$$

where *ρ*_0_ is the standard density at *P*_0_ = 0.1 MPa and *T*_0_ = 298 K, *α* is thermal expansion coefficient, and *β* is compressibility coefficient.

The effects of latent heat *H*_L_^61^ are accounted for by an increased effective heat capacity (${C_{\mathrm{p}}}_{{\mathrm{eff}}}$) and thermal expansion (*α*_eff_) of the partially molten rocks (0 < *M* < 1), expressed as

11 $$\begin{array}{l}{C_{\mathrm{p}}}_{{\mathrm{eff}}} = {C_{\mathrm{p}}} + Q_{\mathrm{L}}\left( {\frac{{\partial M}}{{\partial T}}} \right)_P,\\ \alpha _{{\mathrm{eff}}} = \alpha + \rho \frac{{Q_{\mathrm{L}}}}{T}\left( {\frac{{\partial M}}{{\partial P}}} \right)_T,\end{array}$$

where *C*_p_ is the heat capacity of the solid crust and *Q*_L_ is the latent heat of melting of the crust.

### Model configurations

The overall model setup is based on the tectonic structure of the Himalayan–Tibetan collision zone (Fig. 1), and represents the simplified lithospheric architecture including Neo-Tethyan subduction before the Cenozoic India–Asia collision. According to the stratigraphy and pre-Cenozoic structural configuration of the terranes constituting the Tibetan plateau, a strong terrane is embedded as the integration of the Lhasa and Qiangtang terranes, whereas the adjacent weak terrane represents the combined SPGZ, Kunlun–Qaidam, and Qilian terranes. All the continental plates and terranes consist of a 20-km-thick upper crust, a 15-km-thick lower crust, and an 85-km-thick lithospheric mantle. The oceanic lithosphere is 500-km long and is composed of 8-km-thick crust and 72-km-thick lithospheric mantle. The effects of the potentially thickened crust of the Lhasa terrane (here identified with variable thickness of the left part of the strong terrane; Supplementary Figs 1 and 2) prior to the India–Asia collision are also discussed in the Supplementary Note 2. Different compositions are shown in different colors (Fig. 2a).

Three different rheological profiles of continental lithosphere are designed (Fig. 2b), which represent pro-/retro-plate, strong terrane, and weak terrane, respectively. Flow laws of mafic granulite, plagioclase, and wet quartzite are separately applied for the lower crust of these three lithospheric profiles^58^. In addition, Quartzite and wet quartzite rheologies are used for the upper crust of continental plates and terranes, respectively, whereas the plagioclase rheology for the oceanic crust^58^. Both the lithospheric and asthenospheric mantle are represented by flow law of dry olivine^62^. The sensitivity of model results to the wet-olivine-dominated asthenosphere (Supplementary Fig. 3) has also been discussed in the Supplementary Note 3. Detailed properties of different rock types are described in Supplementary Tables 1, 2.

The model domain has free-slip top boundary and side walls, whereas the lower boundary is treated as mass-conservative permeable to satisfy the free slip of an external boundary at 1000 km below the base of the model. External free slip condition conforms to global conservation of mass in the computational domain, and is implemented by using the following limitation for velocity components at the lower boundary: ∂**v**_*x*_/∂*z *= 0, ∂**v**_*z*_/∂*z *= −**v**_*z*_/Δ*z*_external_, where Δ*z*_external_ is the vertical distance from the lower boundary to the external boundary, where free slip (∂**v**_*x*_/∂*z *= 0, **v**_*z *_= 0) is satisfied. Continental convergence is driven by a constant velocity of 5 cm yr^−1^ assigned in the pro-plate. In order to evaluate the effects of a time-dependent, higher convergence velocity on the terrane deformation behaviors, we also constructed two additional groups of models and discussed the model results (Supplementary Figs. 4 and 5) in the Supplementary Note 4. Generally, a time-dependent, higher convergence velocity probably affects the magnitudes and rates of strain, but would not change the general detachment behaviors of terrane lithospheric mantle during convergence, as well as the resulted overall lithospheric structure.

The initial thermal structure of the continental plate is approximated by two linear interpolations, firstly from the surface (0 °C) to the Moho and then from the Moho to the base of the continental lithosphere (1327 °C). The initial Moho temperature of the two terranes is slightly higher (+50 °C) than that of the pro-plate and retro-plate. The latter are considered as typical stable cratons with a Moho temperature of 400 °C^63^. This makes sense when considering that (1) all these accreted terranes experienced repeated rifting events, oceanic subduction, and continent/arc–continent collision before the Cenozoic Indian collision with Asia^3^, and (2) both the Indian and Tarim–North China lithospheres separated from or amalgamated with other plates acting as stable, strong cratons during the Phanerozoic^64^. The initial thermal structure of the oceanic lithosphere is applied with 0 °C on the surface and 1327 °C at the bottom^63^.

### Model limitations

In the numerical models, the general structures and evolution histories of the India–Asia collision and Tibetan plateau formation agree well with the natural observations. However, some detailed deformation characteristics are not fully resolved, for example, the exact position and activation time of the observed thrust faults, the exact width of the terranes in the plateau, the depth of the underthrusting slabs, and so on. These flaws are secondary and mainly due to the simplifications of the models, for example, the still simplified multi-terrane structure, the neglected erosional variability, and the time-dependent, differential convergence rates. In addition, these 2D numerical models do not involve the out-of-plane deformation (e.g., lateral extrusion, strike-slip faulting, toroidal mantle flow). In the aspect of resulted Himalayan width, we have tried to adjust the detailed parameters in order for the final agreement with nature (see Supplementary Note 5 and Supplementary Fig. 6). It is worth noting that the perfectly consistent model results with observations are not expected in this study, which focuses on the general evolution and controlling factors of the along-strike lithospheric structure of the Tibetan plateau.

### Code availability

The numerical modeling code that supports the finding of this study is available from the corresponding author upon reasonable request.

## Electronic supplementary material

Supplementary Information

## Data Availability

All the relevant data and model output presented in this study are available from the corresponding author upon reasonable request.
