# Supplementary material for: Multi-terrane structure controls the contrasting lithospheric evolution beneath the western and central–eastern Tibetan plateau
Source: Nat Commun. 2018 Sep 17;9:3780. doi: 10.1038/s41467-018-06233-x (PMC6141583; doi:10.1038/s41467-018-06233-x)
Supplement: Supplementary file 1 — Supplementary Information [file 41467_2018_6233_MOESM1_ESM.pdf]

Multi-terrane structure controls the contrasting lithospheric evolution beneath the western and central–eastern Tibetan plateau

Huangfu et al.

Supplementary Note 1

Detailed comparison with previous studies

As is well-known, the Tibetan plateau has typical multi-terrane structure. These major terranes from south to north include Lhasa, Qiangtang, Songpan–Ganzi (SPGZ), Kunlun–Qaidam, and Qilian terranes, with different thermal and rheological profiles according to the stratigraphy and pre-Cenozoic tectonic histories[1]. However, only few previous numerical studies on the Himalayan–Tibetan orogeny took into account the accreted terranes, for example, Kelly et al.[2] (KBB henceforth) is an outstanding and most relevant one. Thus we firstly made a direct and detailed comparison of our work with KBB in the following.

Generally, there are three major differences between KBB and this work. (1) Model setup: KBB considered only one "soft" accreted terrane in the models, which was to represent the Lhasa terrane when applied to the Himalayan–Tibetan system. All the other terranes to the north were not included. We integrated two composite terranes into the model, i.e. the strong and weak terranes, as a first-order simplification, which separately refer to the combined Lhasa–Qiangtang terranes and all the terranes north of the Jinsha suture, according to the stratigraphy and pre-Cenozoic tectonic history of each terrane. Thereby, KBB concentrated on the roles of terrane accretion in the tectonic evolution of southern Tibet, i.e. the Lhasa terrane, whereas our work aims at identifying the effects of different geometric shapes and rheological properties of the terranes on their deformation behavior and the overall lithospheric evolution of the whole plateau. (2) Model results: KBB favored complete detachment of the terrane lithospheric mantle in southern Tibet, which is one of two distinct terrane deformation behaviors revealed by this numerical work. The other one is termed as "partial terrane detachment" mode, in which the weak terrane was first subducted and detached from the retro-plate, resisting the detachment of the strong terrane. The collision ended with subduction of both the pro- and retro-plate beneath the residual terrane. This mode agrees well with the lithospheric structure of central–eastern Tibet revealed by recent geophysical observations[3–5], which was not shown in KBB, owning to different focus. (3) Main conclusion and geological implications: KBB indicated that a strongly metasomatized (i.e., dense and weak) terrane lithospheric mantle tended to be removed, and thus promoted later horizontal underthrusting. It was supposed as a prototype representation of present-day underthrusting of the Indian mantle lithosphere beneath the Lhasa crust. KBB's numerical results directly supported previous conceptual interpretations that the southern Tibetan lithospheric mantle was removed. This study suggests two distinct terrane deformation behaviors in the framework of Tibetan tectonics, which represent the overall tectonic evolution of western and central–eastern Tibet. More importantly, we propose for the first time that it is the geometric shape of the combined Lhasa–Qiangtang terranes that dominates the different terrane behaviors of western and central–eastern Tibet during the India–Asia collision.

Another relevant study is one of our previous works that also integrated an accreted terrane into the model design[6]. That work focused on the mechanism of lithospheric delamination during continental collision, and predicted three basic modes (pro-plate delamination, retro-plate delamination, and transitional double-plates delamination) as well as their favored conditions. We applied the mode of retro-plate delamination to the Himalayan–Tibetan system, and proposed that the thin Tibetan lithosphere was attributed to delamination. Actually, multi-terrane configuration of the Tibetan lithosphere was not included in the model setup of Li et al.[6], which just simply regarded the whole Tibetan plate as a weakened lithosphere. In this aspect, the current work is greatly improved, with consideration of the complex tectonic histories of these terranes comprising the whole plateau (for the first time).

Supplementary Note 2

Influence of the thickened crust of Lhasa terrane before the India–Asia collision

The widely distributed thrusts in the Lhasa and Qiangtang terranes indicate intense upper-crustal shortening from late Cretaceous to early Tertiary[7–10]. Balanced cross-section restoration, together with synthrusting deposition and $^{40}Ar/^{39}Ar$ results, reveals that these thrusts in the Lhasa terrane were activated roughly from late Cretaceous to Paleocene, and accommodated major crustal shortening[11–14]. Thereby, it is considered that the Lhasa terrane was probably thickened prior to the Cenozoic India–Asia collision.

In order to evaluate the effect of the preceding crustal thickening of Lhasa terrane on the India–Asia collision, we have conducted two additional groups of models with thicker crust (40 km and 45 km) in the left part of the strong terrane, representing the thickened Lhasa terrane crust caused by Meso-/Neo-Tethyan oceanic subduction and terrane collision. The model results (Supplementary Figs 1 and 2) show that a larger crustal thickness of the Lhasa terrane can indeed affect the tectonic processes during continental collision (such as crustal thickness of the collision zone, extent of crustal melting, and topographic evolution). However, the main conclusion does not change (cf. Fig. 5c in the main text and Supplementary Fig. 1a), ie., a narrow strong terrane favors complete detachment of the accreted terranes, while a wider strong terrane facilitates its (partial) preservation between two colliding plates. Thereby, the modes of terrane detachment during collision do not depend very much on the initial crustal thickness of the terranes.

Supplementary Note 3

Influence of the wet olivine flow laws for the asthenosphere

In the reference models (Fig. 5c), both the lithospheric and asthenospheric mantle are represented by the flow laws of dry olivine[15]. In order to test the sensitivity of rheological properties of the asthenosphere, we constructed an additional group of models in which the asthenosphere is applied by the flow laws of wet olivine[16].

The model results are summarized in Supplementary Fig. 3. After making comparisons with the model results of the reference group (cf. Fig. 5c and Supplementary Fig. 3a), it suggests that changing the flow laws of the asthenosphere from dry olivine to wet olivine does not affect the model results in terms of terrane detachment behaviors during collision. Similarly, the "complete terrane detachment" mode occurs, when the initial width of the strong terrane is not larger than 800 km; otherwise, the "partial terrane detachment" mode develops.

Supplementary Note 4

Influence of the time-dependent convergence velocity

The reconstructed India–Asian convergence velocity history is still widely debated[17–20]. Generally, two velocity drops are inferred to take place, i.e. from ~15 cm yr^{-1} during Neo-Tethyan subduction to ~10 cm yr^{-1} in the initial collision, then to ~5 cm yr^{-1} during "hard" collision. The convergence velocity at present still reaches 3.5–4 cm per yr[21,22]. Because the evolution of Indian convergence velocity is so complex, applying such a time-dependent velocity in the models is not helpful for better understanding the dynamics of the India–Asia collision. It is the reason why most of previous numerical studies applied an averaged convergence rate of 5 cm per yr[2,23]. Therefore, we also assigned a constant convergence velocity of 5 cm yr^{-1} in the reference models during continental collision (Figs 3–5 in the main text).

In this section, we constructed two additional groups of models in order to evaluate the effects of time-dependent, higher convergence velocities on model results. In the first group, the imposed velocity is assigned to 15 cm yr^{-1} during oceanic subduction, which then reduces to 10 cm yr^{-1} during the first 10 Myrs of continental collision, and eventually drops to 5 cm yr^{-1} for the following collision. This velocity setup is in accordance to Lee and lawver[17]. In the second group, a velocity of 15 cm yr^{-1} is applied during both the oceanic subduction and the first 10 Myrs of collision, which is then decreased to and keeps constant at 5 cm yr^{-1}. The second configuration can be considered as a simplification of the India–Asia convergence rate profile.

The model results of both groups reveal that the increased convergence velocity during preceding oceanic subduction and early-stage continental collision doesn't change the overall conclusion in terms of terrane detachment behavior, although it indeed produces cumulative effects on the tectonic evolution of the collision zone in some aspects, such as the width of the orogenic wedge, the timing of the terrane detachment (Supplementary Figs 4 and 5). Thereby, we think that a constant convergence velocity of 5 cm yr^{-1} in the collision models is valid for this study on Tibetan tectonics, which focuses on the multi-terrane detachment.

Supplementary Note 5

The controlling factors in the width of modeled Himalaya

Previous studies on kinematic plate reconstruction indicated that the post-collisional convergence between India and Asia might reach more than 2000 km[24,25], with ~900-km-long convergence absorbed by the Indian lithosphere[26,27]. The Himalayan belt is generally regarded as the orogenic wedge of the India–Asia collision zone, the development of which was ascribed to off-scraping and accumulation of the detached upper portion of the Indian crust during Indian plate subduction/underthrusting[8]. The Himalaya, with an along-strike length of ~2000 km and an average width of ~300 km, not only plays a critical role in shaping our understanding of orogenesis, but also serves as a direct observational constraint to evaluate model results[28].

As for the reference groups of models, quartzite rheology is applied for the pro-plate upper crust, while wet quartzite for the upper crust of both terranes. The former represents the strong Indian upper crust, and the latter for the relatively weak Tibetan upper crust. Model results show that the resulted Himalaya has a width of ~300 km in the "complete detachment model" mode (Fig. 3e in the main text) and ~400 km in the "partial detachment model" mode (Fig. 4d in the main text). Actually, the width of modeled Himalaya is always < 400 km in all the models (Fig. 5), which are largely consistent with the natural case. Furthermore, ~1000 km of the total post-collisional convergence (~2500 km) is accommodated by the pro-plate, which can also be reconciled with previous geophysical studies[26,27].

In order to explore how the rheological properties of the pro-plate crust regulate the architecture of orogenic wedge, we constructed four additional groups of models with wet quartzite rheology, but variable thicknesses for the pro-plate upper crust. In comparison with the reference group, the model results reveal that modifying the upper crustal rheology or its thickness can clearly affect the width of modeled orogenic wedge (Supplementary Fig. 6a).

First, a decrease of rheological strength of the pro-plate upper crust, through replacing quartzite rheology with wet quartzite rheology, leads to a much wider orogenic wedge, compared to the reference models (cf. Figs 3 and 4 in the main text and Supplementary Fig. 6b,c). The width of orogenic wedge in the models with 20-km-thick, wet quartzite upper crust of the pro-plate reaches more than 600 km (even up to 1000 km) in both the "compete terrane detachment" and "partial terrane detachment" modes, after a post-collisional convergence of 2500 km.

Second, the orogenic wedge significantly narrows with decreasing the upper crustal thickness of the pro-plate, owning to a decreased amount of crustal materials detached from the subducting slab (Supplementary Fig. 6b,c). When the pro-plate upper crustal thickness is decreased to 18 km, 16 km, or 14 km, the resulted Himalaya width is decreased to ~300 km, ~250 km, or ~200 km, respectively (Supplementary Fig. 6b), in the "complete terrane detachment" mode after a 55-Myr collision. Similarly, the

modeled Himalaya width in “partial terrane detachment” mode reduces accordingly to a more realistic value (Supplementary Fig. 6c).

The systematic model results reveal that either an increased rheological strength or a decreased thickness of the pro-plate upper crust can result in the development of modeled Himalaya analogous to nature. Meanwhile, it is also presumed that a large proportion of the Indian upper crust, with the underlying lithospheric mantle, has subducted/underthrusted beneath the plateau, under the above-mentioned convergence partition framework constrained by the tomographic image[27,29]. This interpretation agrees well with Ingalls et al.[30] that suggested large-scale subduction of Indian crust implied by India–Asia mass-balance calculation.

Supplementary Tables

Supplementary Table 1 Viscous flow laws used in the numerical experiments

Symbol	Flow laws	E (kJ mol^{-1})	V (J MPa^{-1} mol^{-1})	n	A_D (MPa^{-n} s^{-1})
A	Wet quartzite	154	8	2.3	3.2×10^{-4}
B	Quartzite	156	8	2.4	6.7×10^{-6}
C	Mafic granulite	445	8	4.2	1.4×10^{4}
D	Plagioclase An_{75}	238	8	3.2	3.3×10^{-2}
E	Dry olivine	532	8	3.5	2.5×10^{4}
F	Wet olivine	470	8	4.0	2.0×10^{3}
G	Molten felsic	0	0	1.0	2.0×10^{-9}
H	Molten mafic	0	0	1.0	1.0×10^{-7}

References are from Kirby[31], Kirby and Kronenberg[32], Ranalli and Murphy[33], Ji and Zhao[34], and Ranalli[16].

Supplementary Table 2 Material properties used in the numerical experiments

Material	State	ρ_0 (kg m^{-3})	Cp (J kg^{-1} K^{-1})	k[a] (W m^{-1} K^{-1})	$T_{solidus}$[b] (K)	$T_{liquidus}$[b] (K)	Q_L (kJ kg^{-1})	H_r (μW m^{-3})	Viscous flow laws[c]	Plastic[d] $\sin(\varphi_{eff})$
Sediments & Terrane upper crust	Solid	2700	1000	K1	TS1	TL1	300	2.0	A	0.15
Pro-/retro-plate upper crust	Solid	2700	1000	K1	TS1	TL1	300	2.0	B	0.15
Pro-/retro-plate lower crust	Solid	3000	1000	K2	TS2	TL2	380	0.5	C	0.15
Strong-terrane lower crust & oceanic crust	Solid	3000	1000	K2	TS2	TL2	380	0.5	D	0.15
Weak-terrane lower crust	Solid	3000	1000	K2	TS2	TL2	380	0.5	A	0.15
Pro-/retro-plate & oceanic lithospheric mantle	Dry	3300	1000	K3	–	–	–	0.022	E	0.60
Terrane lithospheric mantle & asthenospheric mantle	Dry	3300	1000	K3	–	–	–	0.022	E	0.26
Weak zone	Wet	3200	1000	K3	–	–	–	0.022	F	0.06
Sediments & upper crust	Molten	2500	1500	K1	TS1	TL1	300	2.0	G	0.06
Oceanic crust & lower crust	Molten	2500	1500	K2	TS2	TL2	380	0.5	H	0.06
References[e]	–	1,2	–	3	4	4	1,2	1	–	–

[a] K1=$[0.64+807/(T+77)]\exp(0.00004P)$; K2=$[1.18+474/(T+77)]\exp(0.00004P)$; K3=$[0.73+1293/(T+77)]\exp(0.00004P)$.

[b] TS1=$889+17900/(P+54)+20200/(P+54)^2$, at $P<1200$ MPa; or $831+0.06P$, at $P>1200$ MPa. TL1=$1262+0.09P$;
TS2=$973-70400/(P+354)+778\times10^5/(P+354)^2$, at $P<1600$ MPa; or $935+0.0035P+0.0000062P^2$, at $P>1600$ MPa. TL2=$1423+0.105P$.

[c] Parameters of viscous flow laws are shown in Supplementary Table 1.

[d] Strain weakening effect is applied for plastic rheology, in which effective friction coefficient ($\sin(\varphi_{eff})$) decreases with larger strain rate.

[e] References 1–4 are Turcotte and Schubert[35], Bittner and Schmeling[36], Clauser and Huenges[37], and Schmidt and Poli[38], respectively.

Supplementary Figures

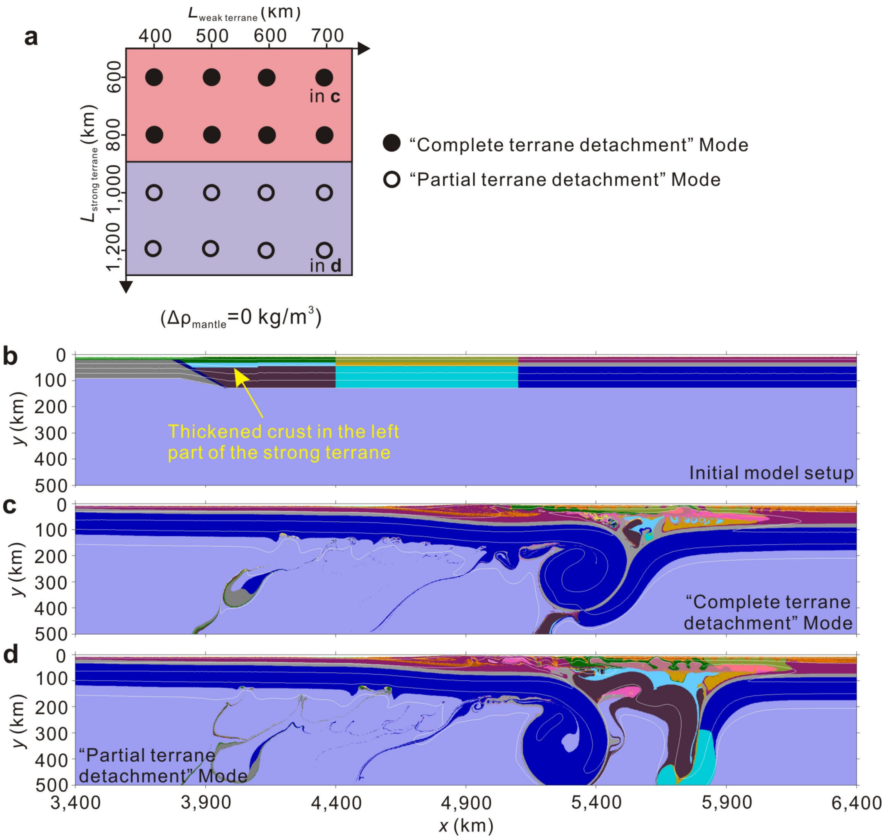

Supplementary Figure 1 Model results with thickened crust (40 km) in the left half part of the strong terrane. (**a**) Regime diagram with 40-km-thick crust in the left half part of the strong terrane and the same reference density between the terranes' lithospheric mantle and the asthenosphere. Two contrasting collision modes are obtained, similar to the reference models (Fig. 5c), which again demonstrates the dependence on the width of the strong terrane. (**b**) Model setup with thickened crust in the left half part of the strong terrane representing the thickened crust of the Lhasa terrane before the Cenozoic India–Asia collision. (**c**) and (**d**) show the typical modes of "complete terrane detachment" and "partial terrane detachment", respectively, both of which are the results at 65 Myr. The corresponding models are marked in the regime diagram in **a**. Colors of rock types are as in Fig. 2a in the main text. White numbered lines are isotherms plotted every 300 °C starting from 100 °C.

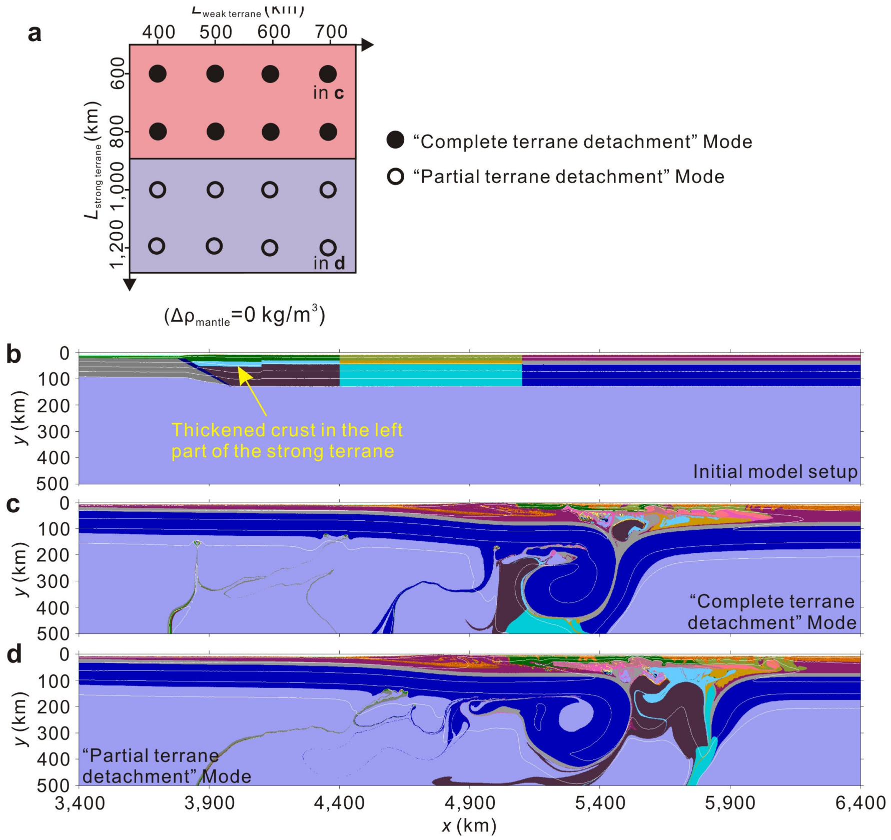

Supplementary Figure 2 Model results with thickened crust (45 km) in the left half part of the strong terrane. (**a**) Regime diagram with 45-km-thick crust in the left half part of the strong terrane and the same reference density between the terranes' lithospheric mantle and the asthenosphere. Two contrasting collision modes are obtained, similar to the reference models (Fig. 5c), which again demonstrates the dependence on the width of the strong terrane. (**b**) Model setup with thickened crust in the left half part of the strong terrane representing the thickened crust of the Lhasa terrane before the Cenozoic India–Asia collision. (**c**) and (**d**) show the typical modes of "complete terrane detachment" and "partial terrane detachment", respectively, both of which are the results at 65 Myr. The corresponding models are marked in the regime diagram in **a**. Colors of rock types are as in Fig. 2a in the main text. White numbered lines are isotherms plotted every 300 °C starting from 100 °C.

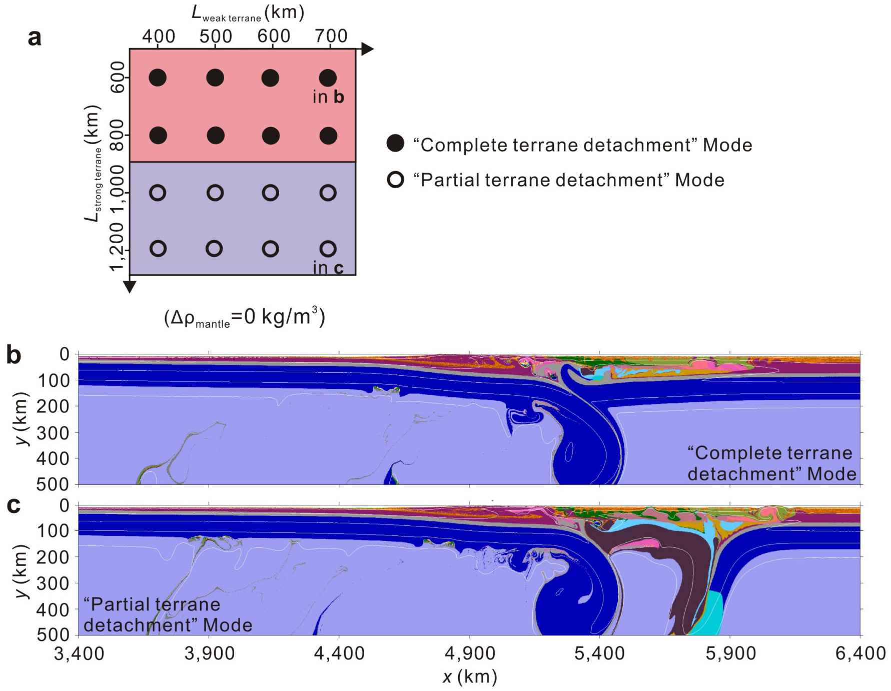

Supplementary Figure 3. Model results with flow laws of wet olivine for the asthenosphere. (**a**) Regime diagram with flow laws of wet olivine for the asthenosphere and the same reference density between the terranes' lithospheric mantle and the asthenosphere. Two contrasting collision modes are obtained, similar to the reference models (Fig. 5c), which again demonstrates the dependence on the width of the strong terrane. (**b**) and (**c**) show the typical modes of “complete terrane detachment” and “partial terrane detachment”, respectively, both of which are the results at 65 Myr. The corresponding models are marked in the regime diagram in **a**. Colors of rock types are as in Fig. 2a in the main text. White numbered lines are isotherms plotted every 300 °C starting from 100 °C.

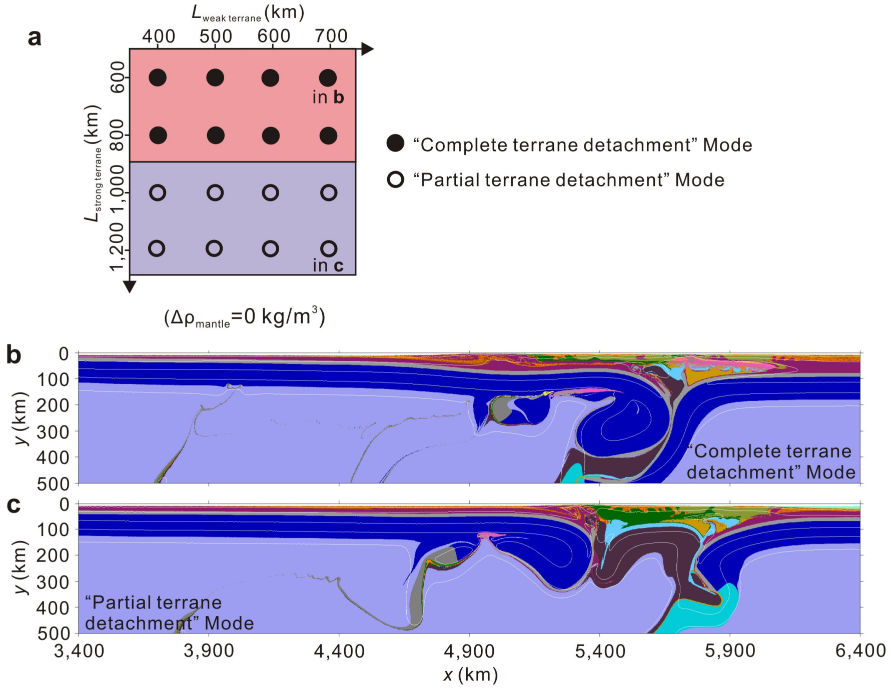

Supplementary Figure 4. Model results with a time-dependent convergence velocity. (**a**) Regime diagram with a convergence velocity of 15 cm yr^{-1} during oceanic subduction, then decreasing to 10 cm yr^{-1} in the first 10 Myrs of continental collision and 5 cm yr^{-1} for the following collision, as well as the same reference density between the terranes' lithospheric mantle and the asthenosphere. Two contrasting collision modes are obtained, similar to the reference models (Fig. 5c in the main text), which again demonstrates the dependence on the width of the strong terrane. (**b**) and (**c**) show the typical modes of "complete terrane detachment" and "partial terrane detachment", respectively, both of which are the results at a convergence of 2500 km during collision. The corresponding models are marked in the regime diagram in **a**. Colors of rock types are as in Fig. 2a in the main text. White numbered lines are isotherms plotted every 300 °C starting from 100 °C.

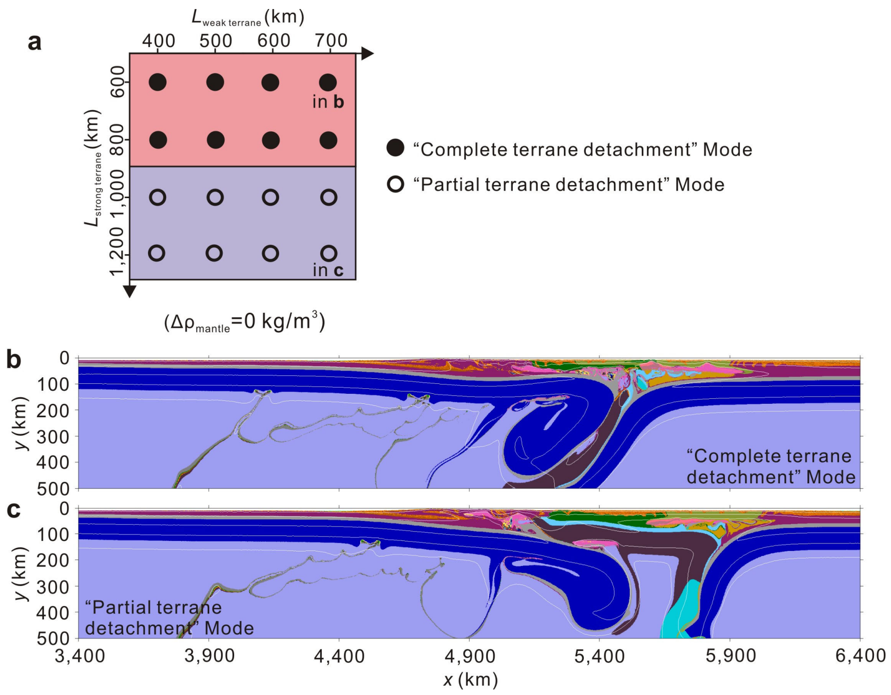

Supplementary Figure 5. Model results with a time-dependent variable convergence velocity. (**a**) Regime diagram with a convergence velocity of 15 cm yr^{-1} during oceanic subduction and the first 10 Myrs of continental collision, then decreasing to 5 cm yr^{-1} for the following collision, as well as the same reference density between the terranes' lithospheric mantle and the asthenosphere. Two contrasting collision modes are obtained, similar to the reference models (Fig. 5c in the main text), which again demonstrates the dependence on the width of the strong terrane. (**b**) and (**c**) show the typical modes of "complete terrane detachment" and "partial terrane detachment", respectively, both of which are the final results at a convergence of 2500 km during collision. The corresponding models are marked in the regime diagram in **a**. Colors of rock types are as in Fig. 2a in the main text. White numbered lines are isotherms plotted every 300 °C starting from 100 °C.

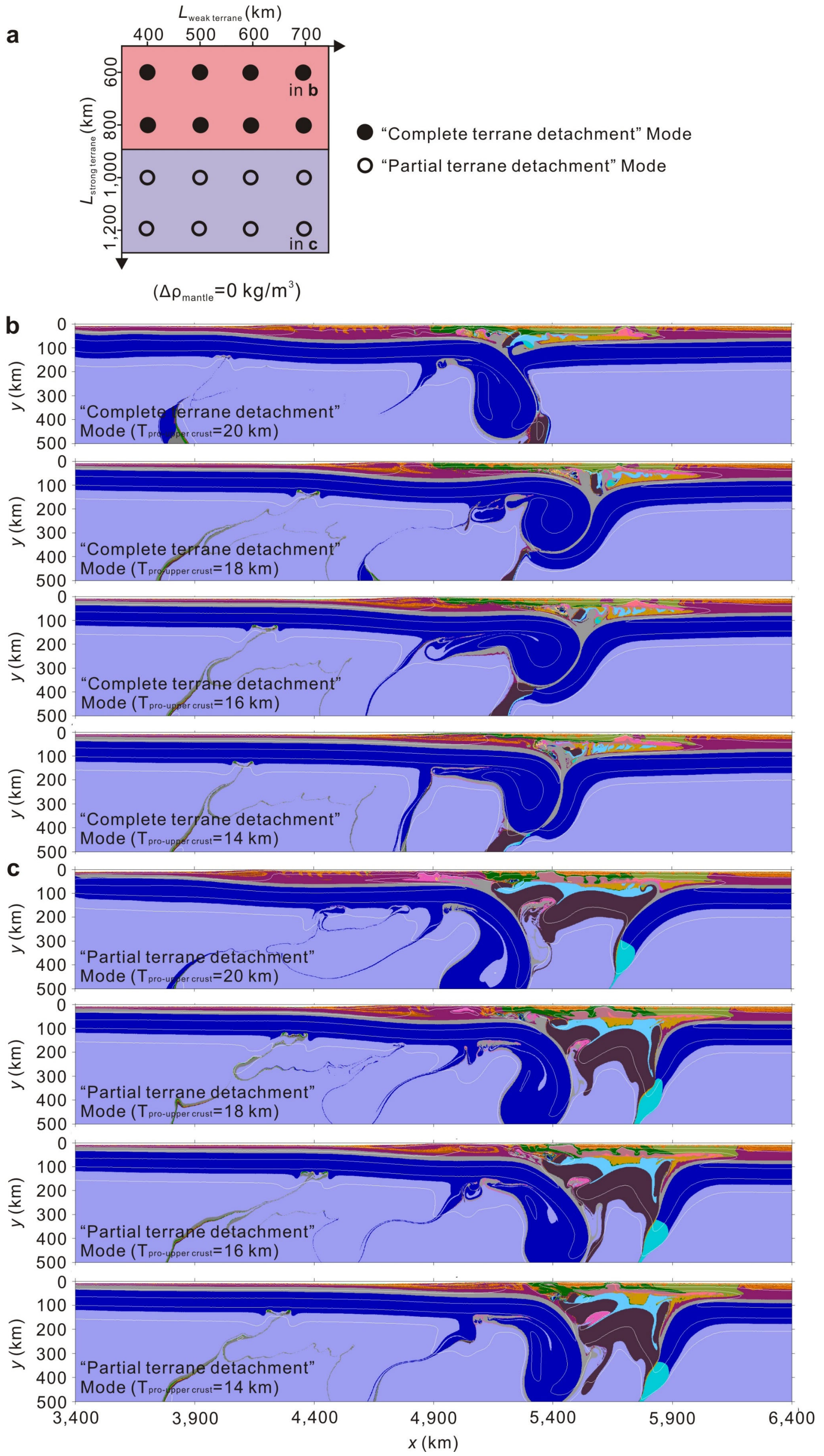

Supplementary Figure 6. Model results with variable thicknesses of the pro-plate upper crust applied by wet quartzite rheology. (a) Regime diagram with variable thickness (20 km, 18 km, 16 km, and 14 km) of pro-plate upper crust and the same reference density between the terranes' lithospheric mantle and the asthenosphere. In contrast to the reference group of models, the pro-plate upper crust in these models is

represented by wet quartzite. Two contrasting collision modes are predicted, similar to the reference models (Fig. 5c), which again demonstrates the dependence on the width of the strong terrane. (b) and (c) show the typical modes of “complete terrane detachment” and “partial terrane detachment”, respectively, all of which are the results at 65 Myr. The corresponding models are marked in the regime diagram in a. Colors of rock types are as in Fig. 2a in the main text. White numbered lines are isotherms plotted every 300 °C starting from 100 °C.

Supplementary References

1. Yin, A. & Harrison, T. M. Geologic evolution of the Himalayan–Tibetan orogen. *Annu. Rev. Earth Planet. Sci.* **28**, 211–280 (2000).
2. Kelly, S., Butler, J. P. & Beaumont, C. Continental collision with a sandwiched accreted terrane: Insights into Himalayan–Tibetan lithospheric mantle tectonics? *Earth Planet. Sci. Lett.* **455**, 176–195 (2016).
3. Ye, Z.*, et al.* Seismic evidence for the North China plate underthrusting beneath northeastern Tibet and its implications for plateau growth. *Earth Planet. Sci. Lett.* **426**, 109–117 (2015).
4. Zhao, J.*, et al.* The boundary between the Indian and Asian tectonic plates below Tibet. *Proc. Natl. Acad. Sci. U.S.A.* **107**, 11229–11233 (2010).
5. Kosarev, G.*, et al.* Seismic Evidence for a Detached Indian Lithospheric Mantle Beneath Tibet. *Science* **283**, 1306–1309 (1999).
6. Li, Z.-H., Liu, M. & Gerya, T. Lithosphere delamination in continental collisional orogens: A systematic numerical study. *J. Geophys. Res.* **121**, 5186–5211 (2016).
7. Ding, L. & Zhong, D. L. Metamorphic characteristics and geotectonic implications of the high-pressure granulites from Namjagbarwa, eastern Tibet. *Sci. China* **42**, 491–505 (1999).
8. DeCelles, P. G., Robinson, D. M. & Zandt, G. Implications of shortening in the Himalayan fold–thrust belt for uplift of the Tibetan Plateau. *Tectonics* **21**, 1062 (2002).
9. Wang, C.*, et al.* Outward-growth of the Tibetan Plateau during the Cenozoic: A review. *Tectonophysics* **621**, 1–43 (2014).
10. Li, Y.*, et al.* Propagation of the deformation and growth of the Tibetan–Himalayan orogen: A review. *Earth Sci. Rev.* **143**, 36–61 (2015).
11. Murphy, M. A.*, et al.* Did the Indo–Asian collision alone create the Tibetan plateau? *Geology* **25**, 719–722 (1997).
12. Kapp, P.*, et al.* Mesozoic and Cenozoic tectonic evolution of the Shiquanhe area of western Tibet. *Tectonics* **22**, (2003).
13. Kapp, P., Yin, A., Harrison, T. M. & Ding, L. Cretaceous–Tertiary shortening, basin development, and volcanism in central Tibet. *Geol. Soc. Am. Bull.* **117**, 865–878 (2005).
14. Volkmer, J. E., Kapp, P., Guynn, J. H. & Lai, Q. Cretaceous–Tertiary structural evolution of the north central Lhasa terrane, Tibet. *Tectonics* **26**, TC6007 (2007).
15. Burov, E. & Watts, A. The long-term strength of continental lithosphere: "jelly sandwich" or "crème brûlée"? *GSA today* **16**, 4 (2006).
16. Ranalli, G. *Rheology of the Earth*. 2nd edn (Chapman & Hall, 1995).
17. Lee, T.-Y. & Lawver, L. A. Cenozoic plate reconstruction of Southeast Asia. *Tectonophysics* **251**, 85–138 (1995).

18. Müller, R. D., Sdrolias, M., Gaina, C. & Roest, W. R. Age, spreading rates, and spreading asymmetry of the world's ocean crust. *Geochem. Geophys. Geosyst.* **9**, Q04006, (2008).
19. Molnar, P. & Stock, J. M. Slowing of India's convergence with Eurasia since 20 Ma and its implications for Tibetan mantle dynamics. *Tectonics* **28**, TC3001, (2009).
20. van Hinsbergen, D. J. J., Steinberger, B., Doubrovine, P. V. & Gassmöller, R. Acceleration and deceleration of India–Asia convergence since the Cretaceous: Roles of mantle plumes and continental collision. *J. Geophys. Res.* **116**, B06101 (2011).
21. Wang, Q.*, et al.* Present-day crustal deformation in China constrained by global positioning system measurements. *Science* **294**, 574–577 (2001).
22. Zhang, P.-Z.*, et al.* Continuous deformation of the Tibetan Plateau from global positioning system data. *Geology* **32**, 809–812 (2004).
23. Magni, V., Allen, M. B., van Hunen, J. & Bouilhol, P. Continental underplating after slab break-off. *Earth Planet. Sci. Lett.* **474**, 59–67 (2017).
24. Molnar, P., England, P. & Martinod, J. Mantle Dynamics, Uplift of the Tibetan Plateau, and the Indian Monsoon. *Rev. Geophys.* **31**, 357–396 (1993).
25. Rowley, D. B. Minimum age of initiation of collision between India and Asia north of Everest based on the subsidence history of the Zhepure Mountain section. *J. Geol.* **106**, 229–235 (1998).
26. Patriat, P. & Achache, J. India–Eurasia collision chronology has implications for crustal shortening and driving mechanism of plates. *Nature* **311**, 615–621 (1984).
27. Replumaz, A., Negredo, A. M., Guillot, S. & Villaseñor, A. Multiple episodes of continental subduction during India/Asia convergence: Insight from seismic tomography and tectonic reconstruction. *Tectonophysics* **483**, 125–134 (2010).
28. Kohn, M. J. Himalayan Metamorphism and Its Tectonic Implications. *Annu. Rev. Earth Planet. Sci.* **42**, 381–419 (2014).
29. Replumaz, A., Guillot, S., Villaseñor, A. & Negredo, A. M. Amount of Asian lithospheric mantle subducted during the India/Asia collision. *Gondwana Res.* **24**, 936–945 (2013).
30. Ingalls, M., Rowley, D. B., Currie, B. & Colman, A. S. Large-scale subduction of continental crust implied by India–Asia mass-balance calculation. *Nat. Geosci.* **9**, 848–853 (2016).
31. Kirby, S. H. Rheology of the Lithosphere. *Rev. Geophys.* **21**, 1458–1487 (1983).
32. Kirby, S. H. & Kronenberg, A. K. Rheology of the lithosphere: Selected topics. *Rev. Geophys.* **25**, 1219–1244 (1987).
33. Ranalli, G. & Murphy, D. C. Rheological stratification of the lithosphere. *Tectonophysics* **132**, 281–295 (1987).

34. Ji, S. C. & Zhao, P. L. Flow Laws of Multiphase Rocks Calculated from Experimental-Data on the Constituent Phases. *Earth Planet. Sci. Lett.* **117**, 181–187 (1993).
35. Turcotte, D. L. & Schubert, G. *Geodynamics*. 2nd edn (Cambridge University Press, 2002).
36. Bittner, D. & Schmeling, H. Numerical Modeling of Melting Processes and Induced Diapirism in the Lower Crust. *Geophys. J. Int.* **123**, 59–70 (1995).
37. Clauser, C. & Huenges, E. Thermal conductivity of rocks and minerals. In: Ahrens, T.J. (Ed.), Rock Physics and Phase Relations: A Handbook of Physical Constants. In: *AGU reference shelf* **3**, 105–126 (1995).
38. Schmidt, M. W. & Poli, S. Experimentally based water budgets for dehydrating slabs and consequences for arc magma generation. *Earth Planet. Sci. Lett.* **163**, 361–379 (1998).
